# Cancer knowledge and awareness among the university students in a high cancer incidence and under-resourced state in northeast India

**DOI:** 10.3332/ecancer.2025.1975

**Published:** 2025-08-26

**Authors:** Vanlaltluangi, Lalengkimi Ralte, John Zothanzama, Lalrammawia Tochhawng, Ruby Zothankimi Ralte, Harvey Vanlalpeka, Nachimuthu Senthil Kumar

**Affiliations:** 1 Department of Education, Mizoram University (A Central University), Aizawl 796004, Mizoram, India; 2 Department of Biotechnology, Mizoram University (A Central University), Aizawl 796004, Mizoram, India; 3 Department of Nursing, Regional Institute of Paramedical and Nursing Sciences, Zemabawk, Aizawl 796017, Mizoram, India; 4 Department of Obstetrics and Gynaecology, Zoram Medical College, Falkawn, Aizawl 796005, Mizoram, India

**Keywords:** knowledge, awareness, cancer, university students, information sources, intervention strategies

## Abstract

**Purpose:**

This study aims to explore the knowledge regarding cancer causes and prevention among university students. Research on cancer awareness and comprehension is vital for promoting both individual and public health, particularly in regions with high cancer prevalence.

**Methods:**

A self-administered questionnaire survey was used to assess the comprehension and knowledge of cancer. The sum of sample was 756 university students from various departments who participated in the survey. Data were analysed using Microsoft Excel and SPSSv.20 programs. Statistical significance was determined using chi-square tests and p-values.

**Results:**

The student’s provenance of cancer education were reported as 20% from both friends or family and social media. Barely, 19.68% felt confident about their knowledge of cancer, while 33.8% of students preferred cancer awareness and campaigns as their primary source of learning about cancer. Overall, 59.5% of students demonstrated some level of knowledge related to Cancer. The association between student’s knowledge and awareness on cancer education scores was statistically significant (p = 0.000).

**Conclusion:**

The majority of students perceive unassured in their knowledge of cancer and hence, strengthening awareness campaigns and educational initiatives is crucial for promoting early detection and prevention, enhance the quality of treatment and reduce stigma. Furthermore, there is a dearth of information on the high-risk human papillomavirus vaccine and individuals should be made aware of the significance of getting vaccinated as a preventive step.

## Introduction

Mizoram has the highest cancer rate in terms of population in India. The region had 1.25 million people in 2023, and when compared to other northeastern regions, Mizoram’s

Aizawl district had the greatest incidence of stomach cancer [1]. Mizoram, a northeastern Indian state, is classified as a lower-middle-income region by the World Bank.

According to the Population-Based Cancer Registry, the age-adjusted incidence rate of cervical cancer (CC) varies greatly among registries; the highest is 23.07 per 100,000 in Mizoram state [2]. The International Agency for Research on Cancer reports in 2023, there were approximately 127,526 new cases of CC in India, with an age-standardised incidence rate of 17.7 per 100,000 women. In the same year, an estimated 79,906 women died from the condition, for an age-standardised death rate of 11.2 per 100,000 women [3]. Among all gynaecological diseases, CC ranks among the most prevalent causes of morbidity and death globally. Most healthcare workers in the workforce lack appropriate knowledge about CC [4]. Indian women tend not to be particularly conscious of CC and how they can prevent it. A quick and affordable method for detecting CC in its initial phases is the Pap test [5].

Despite its small size, Mizoram has made significant efforts to improve its healthcare infrastructure, particularly through government-sponsored programs and community health initiatives. However, obstacles exist in cancer prevention, particularly CC, which remains a major public health concern. CC screening is accessible through government health facilities, although coverage is restricted, especially in rural regions. These factors contribute to the ongoing CC burden in the state, emphasising the need for increased knowledge, access and legislative action [6].

Persistent infection with high-risk human papillomavirus (HPV) types is the leading cause of CC. HPV types 16 and 18 account for over 70% of CC cases globally. In India, the prevalence of HPV among women with invasive CC can approach 98%, with HPV type 16 accounting for more than 90% of the cases [7, 8]. CC prevention consists of two main strategies: primary prevention via. vaccine and secondary prevention through screening programs. In September 2022, India launched Cervavac, a home-made product. Despite the availability of these vaccines, acceptance has been limited due to cost, low awareness and cultural hurdles. To combat this, the Indian government has decided to prevent CC by vaccinating girls, with the goal of incorporating HPV vaccine into the Universal Immunisation Programme to improve coverage and accessibility [9]. Secondary prevention of CC includes screening and treatment if requested. The goal of screening is to detect and treat patients who show early indicators of sickness, usually by the use of a low-cost, precise and reliable test that is widely applicable. Another goal of screening is to reduce CC-related deaths by detecting the disease at an early stage when it is still treatable or by detecting precursor lesions [10]. CC has a substantially higher frequency and burden among women with low socioeconomic status (SES) and rural women in India. Unlike in industrialised countries, CC prevention programs in poor countries have failed to reach their goals due to financial, societal and logistical constraints. Furthermore, the high incidence of CC can be lowered with the adoption of a nationwide HPV vaccination program, but the high cost for the vaccinations makes it difficult to administer in an under-resourced state like Mizoram [11].

In Mizoram, the proportion of women aged 30–49 years who have undergone screening recorded as 2.7% for breast cancer, oral cancer at 0.9% and CC at 6.9%. Of the men aged 30–49, just 1.2% had previously screening for oral cancer. Men were most likely to have cancer in the Aizawl district of Mizoram (age-adjusted rate of 269.4 per 100,000) [12]. Breast cancer is one of the most common malignant disease and accounts for 23% of cancer-related deaths in postmenopausal women. This is a worldwide problem now, but because women are so careless when it comes to self-examination and professional breast examinations, the disease is still detected in its advanced stages [13, 14]. In contrast to the diagnosis of breast malignancy, the general public knows relatively little about cancer of the colorectal. Colorectal cancer (CRC) is a major issue for social and civilisation [15]. This emphasises how crucial civic education is in combating this widespread cancer [16]. Diabetes must be made widely known to the public as a significant risk factor for CRC. Public lectures, school visits and online forums will all play a significant part in informing the public about the signs and methods of CRC diagnosis [17].

Use of tobacco in any way, including chewing and smoking (tuibur and smokeless), raised Mizoram’s susceptibility to stomach cancer [18]. The risk factor associated with meizial (local cigarettes) smoking was greater than that of other tobacco-related behaviours. The incidence of stomach cancer was enhanced by cigarettes, betel nut use and tuibur (water laced with tobacco smoke) [19]. The significant contribution of wood smoke, cooking oil emissions, eating smoked meat, smoked fish and soda (an alkali preparation used as food additions in Mizoram) led to the increased risk of lung cancer in Mizoram women. A substantial protective effect was noted for the consumption of eggs and bamboo shoots [20]. Frequent eating of smoked dry salted meat and fish, as well as sa-um, a traditional dish made from fermented pork fat, has been linked to an increased risk of stomach cancer [21]. The likelihood that a person would have a cancer test and treatment was still influenced by sociocultural variables, education and access to medical facilities. Therefore, in order to reach the public, especially the rural population, health communications and information, as well as campaigns to raise awareness of cancer screening, should be strengthened. These programs and efforts have the potential to improve rural residents’ and vulnerable population members’ access to cancer knowledge about cancer screening programs and treatment facilities [22]. Since those in higher SES are prone to obtaining new information at a quicker pace than lower socioeconomic strata groups, increased information flow frequently results in widening knowledge gaps across various socioeconomic groups. These information gaps might provide a strong but incomplete explanation for the differences in risk-taking habits and health outcomes between various social groups [23]. Preventive and prompt diagnosis of the illness can be aided by being aware of risk variables and early indicators in the population as a whole [24]. Low awareness might result in a delayed diagnosis and treatment that begins too late, which is one of the main causes of the high death rates from cancer.

By teaching people about the symptoms that are indicative of various cancer kinds, cancer awareness works to promote early detection. Inaccurate knowledge as well as social stigma related to cancer may prevent people from getting timely medical care. Adopting healthy lives is emphasised as a key component of cancer awareness programs. A person’s ability to take control of their health and embrace behaviours that may lower their chance of cancer is enhanced by their awareness of risk factors, proactive measures and accessible screening alternatives. Initiatives to raise awareness help people who are impacted by cancer feel connected to one another and supportive of one another. For both the individual and their family, receiving a diagnosis of cancer can have an enormous psychological effect. The mental health issues related to malignancy and its therapeutic management can be addressed with knowledge of the assets and assistance services that are accessible [25].

The public’s perceptions and understanding of health issues, such as cancer, are greatly influenced by the media. An individual’s knowledge and awareness of cancer can be greatly influenced by traumatic events such as experiencing a relative or friend who has been diagnosed with the disease. People who experience cancer firsthand may be more inclined to learn about the disease and take preventative action. For instance, various ages or genders may benefit more from different cancer screening guidelines. Information about health can be shared through interpersonal relationships and community networks. Peer relationships, support groups and initiatives in the community can all help spread information and increase awareness of cancer [25].

In the present study, cancer education, specifically the knowledge and awareness of various types of cancers have been examined among University students. The research focuses on Mizoram state in Northeast India, which is an under-resourced state that has reported a high incidence of cancer cases in recent years. Given the alarming prevalence of cancer in the region, there is a critical need to evaluate the existing level of understanding among the youth, who play a crucial role in future public health initiatives.

## Material and methods

### Study design

This study employed a descriptive research design using a self-administered questionnaire to assess the knowledge, understanding, attitude and perceptions related to cancer education among the students of Mizoram University.*Study subjects and study settings*

The study was conducted at Mizoram University, located in Aizawl, the capital of Mizoram. University students from various academic departments were invited to participate. The survey was conducted in both online and offline formats to maximise reach and convenience.

- Inclusion criteria: All enrolled undergraduate and postgraduate students of Mizoram University who identified as Mizo tribe and consented to participate were included in the study.
- Exclusion criteria: Students who did not provide informed consent were excluded from the study.

### Study instrument

The primary tool for data collection was a structured, self-administered questionnaire developed in English and translated into the Mizo language to ensure better comprehension. The questionnaire consisted of socio-demographic information, Sources and perception of cancer education, lifestyle habits, preferred methods of receiving cancer education, Knowledge assessment quiz and attitude-related questions. The knowledge section included statements with response options of ‘True,’ ‘False’ and ‘I don’t know,’ while the attitude section used a 3-point Likert scale (‘Agree,’ ‘Neither agree nor disagree’ and ‘Disagree’).

### Study variables

The Independent variables were age, gender, academic department, level of education and lifestyle habits. In addition, the dependent variables were knowledge score, attitude score and preferences for cancer education.

### Ethical considerations

Ethical approval for the study was obtained from the Institutional Ethics Committee of Mizoram University (MZU/IHEC/2015/007). Informed consent was obtained from all participants prior to their participation. Confidentiality and anonymity were assured and participation was entirely voluntary.

### Statistical analysis

Data were collected both online (via Google Forms) and offline (through printed questionnaires). Microsoft Excel was used to compile and organise the responses. Statistical analysis was conducted using SPSS version 20. Descriptive statistics such as frequency and percentage were used to summarise the data. The chi-square test was applied to examine associations between socio-demographic variables and cancer knowledge. A *p*-value of less than 0.05 was considered statistically significant.

## Results

**Figure 1.**
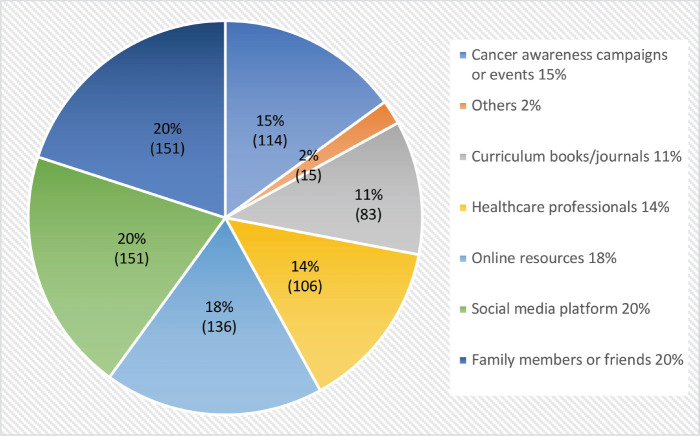
Sources of cancer education among the university students (*n* = 756).

**Figure 2.**
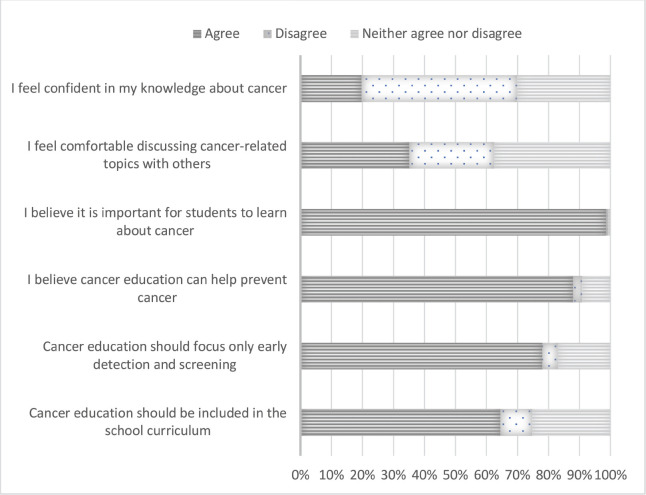
Perception of cancer education among university students (*n* = 756).

From the response, about 55.7% of the 756 responders are female and 44.3% are male. The respondents’ age ranged from 18 to 33 years old, with an average age of 23. According to Figure 1, 20% of the students’ sources of cancer education came from friends or family and social media sites combined. This is followed by 18% from online resources, 15% from cancer awareness events or campaigns, 14% from healthcare providers, 11% from textbooks and journals and 2% from other sources, including cigarette covers. Students’ perceptions of cancer education are depicted in Figure 2, where 64.52% of them agrees on its inclusion in the curriculum while 10.17% disagrees. About 78.05% of students support emphasising early detection and screening in cancer education, compared to 4.95% who disagree. However, 87.95% think that cancer education can prevent cancer. Also, 98.53% of students think it’s important to learn about cancer, compared to 0.54% who disagree and 35.07% of students feel comfortable talking to others about cancer-related topics. Only 19.68% of students felt sure about their knowledge about cancer, whereas 50.20% disagreed.

**Figure 3.**
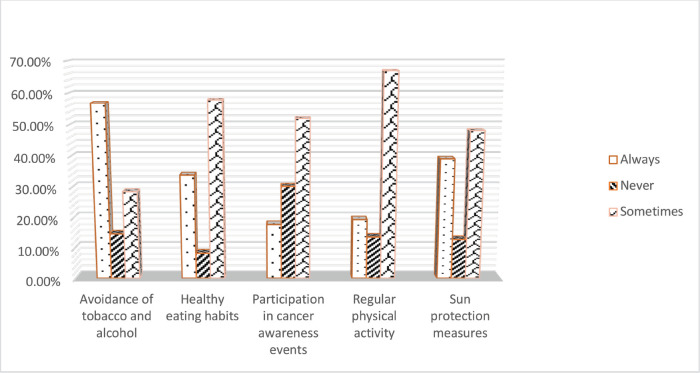
Knowledge about cancer-related lifestyle habits among the university students (*n* = 756).

**Supplementary Figure 1.**
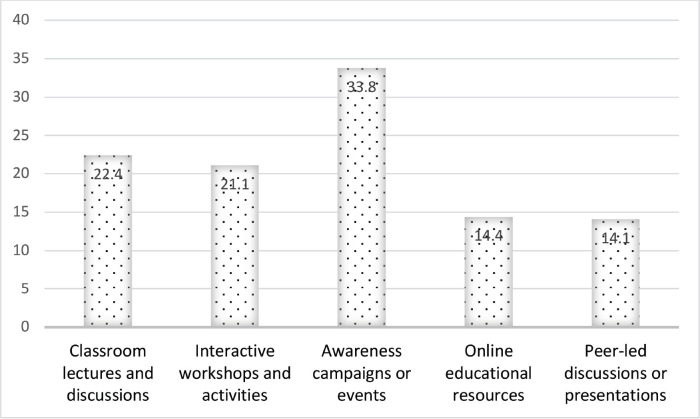
Perception among the university students about the preferred cancer education methods (in percentage, *n* = 760).

Figure 3 illustrates a lifestyle linked to cancer: 56.76% of students constantly abstain from alcohol and tobacco, compared to 14.73% who never do so and 28.51% who do so occasionally. 33.87% of students follow a healthy diet, 8.30% never do and 57.83% occasionally do. 30.25% never and 51.94% occasionally participate in cancer awareness events, compared to 17.80% who do so often. 19.41% of students engaged in physical activity consistently, 13.65% never did so and 66.93% did so occasionally. While 39.09% of students constantly use sun protection measures, 12.85% of students never use them and 48.06% of students use them occasionally. Supplementary Figure 1 illustrates the various ways that students preferred to learn about cancer. Of these, 33.8% preferred awareness campaigns or events, followed by lectures and discussions in the classroom (22.4%), interactive workshops and activities (21.1%), online educational resources (14.4%) and peer-led discussions or presentation techniques (14.1%). A thorough summary of the students’ cancer education information is given in Table 1. Of the students, 59.5% possess knowledge about cancer education, while 40.5% lack it. While 70.8% of the students were conscious of how we come in contact with carcinogens and 43.16% students were enlightened on some of the information we need to determine if a substance might cause cancer. The student’s knowledge and awareness on cancer education scores were highly significant (*p* = 0.000).

## Discussion

### Rationale of the study

Cancer remains a leading cause of morbidity and mortality worldwide, with growing concern about its rising incidence in low- and middle-income regions such as Mizoram. In this context, improving awareness and preventive behaviours—especially among youth—can significantly contribute to early detection and better health outcomes. University students represent an ideal target group for such interventions as they can become agents of change in their communities. This study was conducted to assess the understanding, knowledge and attitudes of university students at Mizoram University regarding cancer education and prevention.

### Main findings of the study

The study found that most students had a basic awareness of cancer, but only 19.68% felt they knew enough about the disease. A significant 98.53% believed that it is important to educate children about cancer and many agreed that awareness can aid in prevention. Notably, only 35.07% felt comfortable discussing cancer-related topics openly. Approximately 78% of students supported prioritising early screening and detection, and while 33.39% claimed to always lead a cancer-preventive lifestyle (abstaining from tobacco/alcohol, exercising and healthy diet), 50.66% did so only occasionally. Social media, family and friends were cited as the primary sources of information, while awareness events and classroom education were preferred methods for learning about cancer.

### Distinctive features of this study

This study is one of the few investigations focusing specifically on cancer awareness and lifestyle practices among Mizo youth at the university level. It is unique in capturing both knowledge levels and preferred educational methods within a localised cultural and social context, including the influence of community-based organisations such as the Young Mizo Association (YMA). Furthermore, the study employed both online and offline methods to ensure inclusivity and broad representation from various academic departments.

### Advancement of current understanding

Previous studies have emphasised the role of awareness in the prevention of cancers, especially those preventable through vaccination and early screening, such as CC. This study reinforces that despite general awareness, there are substantial knowledge gaps, particularly regarding HPV vaccination, the link between diet and cancer and sun protection practices. It also highlights that while students are socially active and open to learning through peer and digital platforms, structured and culturally tailored educational initiatives remain limited.

### Subject of the discussion

Cancer education in Mizoram remains insufficiently developed and highly fragmented. Findings from the present study indicate that university students primarily acquire cancer-related information through informal sources, such as family, friends and social media platforms. This reliance on non-institutional sources highlights a significant gap in structured, school-based cancer education initiatives. Given the influential role of community organisations such as the YMA, these entities present an important opportunity to extend and strengthen community-based health promotion efforts. Students’ preference for awareness campaigns and teacher-led discussions further underscores the potential effectiveness of incorporating cancer education into formal academic curricula.

Parallel observations have been reported in a study conducted in Kazakhstan, where low baseline knowledge and confidence regarding cancer were noted among both students and mothers. In Kazakhstan, only approximately 20% of students expressed confidence in their cancer knowledge and HPV awareness among mothers was nearly absent unless they possessed pre-existing positive attitudes toward vaccination. Statistical analysis revealed a strong correlation between education and favourable attitudes (*p*  < 0.005 for mothers; *p*  =  0.000 for students), suggesting that structured educational interventions may significantly improve health outcomes. These findings advocate for national-level awareness campaigns for parents and the use of social media to engage students—strategies that may be adaptable to Mizoram’s sociocultural context. Further insights emerge from international comparisons. A multi-country survey assessing HPV awareness in the United States, the United Kingdom and Australia reported the highest levels of awareness in the US (88%), followed by Australia (72%) and the UK (62%). Across all three settings, men and individuals with lower educational attainment consistently demonstrated poorer awareness levels. Women in the US and UK outperformed men on a 15-item HPV knowledge test (mean scores ≈9.2 and 8.4, respectively), while Australian gender differences were less pronounced. Moreover, 79% of respondents familiar with HPV reported awareness of the HPV vaccine, with US women (92%) showing the highest levels of knowledge compared to their UK and Australian counterparts (both 81%). These international data emphasise the role of both education and targeted public health strategies in enhancing cancer and HPV-related awareness [26, 27].

In response to the recognised need for improved prevention efforts, the Health and Family Welfare Department of Mizoram announced in January 2025 the introduction of a quadrivalent HPV vaccine program for girls aged 9–14 years, provided free of cost [28]. The vaccination program will primarily be delivered through school-based sessions, supplemented by catch-up clinics at health facilities and targeted outreach to out-of-school adolescents. Implementation strategies include integration with existing adolescent and school health programs, cold-chain infrastructure assessments and real-time data management via the U-WIN application. Collaborative initiatives with the Cancer Foundation of India have already resulted in a successful demonstration project in Aizawl district, where approximately 20,000 girls aged 9–13 years were vaccinated. Notably, local authorities actively engaged in public lectures to raise community awareness regarding HPV and CC prevention [29, 30].

The study also highlights several lifestyle-related risk factors prevalent among youth in Mizoram, including frequent consumption of smoked foods, use of indigenous tobacco products such as *zozial* and limited awareness of the risks associated with excessive sun exposure. These behaviours contribute to the region’s elevated cancer burden and necessitate targeted educational and behavioural interventions.

Despite the availability and proven efficacy of HPV vaccination in preventing CC, awareness and vaccine uptake among university students remain low, signaling an urgent need for comprehensive health promotion campaigns and supportive policy measures. While education alone may not fully eradicate cancer, increasing knowledge and awareness has the potential to significantly influence preventive behaviours, promote early detection and improve health-seeking practices.

In conclusion, the findings of this study support the urgent implementation of integrated, community- and school-based cancer education initiatives. A multi-sectoral approach that combines public health infrastructure, formal education systems and community organisations offers a promising pathway toward reducing the cancer burden in Mizoram and similar under-resourced settings.

### Agreement and disagreement

Our study agrees with findings that link lifestyle choices—such as tobacco use, alcohol consumption and diet—to increased cancer risks. It also echoes research from similarly developing nations that suggests young people are interested in learning about cancer but lack proper channels. However, it diverges in noting a particularly strong preference for community-based approaches and peer education through organisations like YMA, which is less commonly emphasised in global studies.

In summary, this study highlights a clear demand and necessity for enhanced cancer education in Mizoram, particularly among university students who can serve as change agents in their communities. It underlines the importance of integrating cancer-related topics into school curricula and organising awareness campaigns that include local organisations such as the YMA.

This study has several strengths, including its focus on a region with unique cultural and lifestyle practices influencing cancer risk, and its examination of multiple factors—knowledge, attitudes, behaviours and preferred educational methods. It also brings attention to the significant role that informal networks like family and social media play in spreading cancer awareness.

Despite these limitations, the study is primarily linked to its descriptive research design, which does not allow for the establishment of causal relationships between variables. The study describes the current state of knowledge and practices, but cannot determine the reasons behind low awareness or predict future behaviour. Additionally, the study was conducted within a single university setting, which may limit the generalisability of the findings to all young people in Mizoram. Self-reported data may also be subject to response biases, such as over- or under-reporting certain behaviours.

## Conclusion

The study revealed that it is important for the students to know about the basic knowledge of cancer and organising awareness and prevention programmes by the schools, governments and some non-profit organisations, such as YMA and so on, since the majority of students were not confident in their knowledge. Also, the data offers new insight into the knowledge of cancer vaccination, such as HPV to be stressed on awareness, as most students do not know what it is about. The source of cancer education was mostly from social media and family or friends. The study concludes that giving awareness through social media should be emphasised. Awareness can also be done by giving out posters, having discussions in the classrooms guided by the teacher on how we can prevent different diseases. The way we live defines how we come in contact with different problems, so eating healthy foods and physical activity assist in solving many problems. In Mizoram, eating processed foods, soda, smoking zozial, eating betelnuts, tuibur and drinking cause many health issues. Awareness should be prioritised.

## Conflicts of interest

The authors have no conflicts of interest.

## Funding

No funding was received for this study.

## Consent to participate

Written consents were obtained from the participants.

## Ethical approval

This work was approved by Mizoram University Institutional Human Ethical committee (MZU/HEC/2024/006), dated: 16.01.2024.

## Availability of data and material

The raw data can be made available on reasonable request for research purposes.

## Author contributions

VT did data collection, analysis and paper writing; LR did statistical analysis, paper writing; JZ, LT, RZR, HV, NSK conceptualised the study, funding resources, data management. All the authors edited and approved the final draft of the manuscript.

## Figures and Tables

**Table 1.** Frequency of participants with knowledge and awarness of cancer (*n* = 756).

Variables	Categories	Frequency (%)	Chi square value	*p* value
Cancer cells rob nutrients from nearby tissues and destroy normal cells or push them out of the way	False	13 (1.7%)	467.008^a^	0.000
	True	498 (65.9%)		
	I don’t know	245 (32.4%)		
Cancer can only be definitely diagnosed by looking at cells or tissue under a microscope	False	175 (23.1%)	43.675^a^	0.000
	True	258 (34.1%)		
	I don’t know	323 (42.7%)		
Stage of cancer tells us how far the cancer has spread	False	24 (3.2%)	1091.937^a^	0.000
	True	680 (89.9%)		
	I don’t know	52 (6.9%)		
A positive cancer diagnosis means that you are for swear going to die of cancer	False	635 (84.0%)	874.024^a^	0.000
	True	50 (6.6%)		
	I don’t know	71 (9.4%)		
Cancer can occur anywhere in the body	False	50 (6.6%)	738.913^a^	0.000
	True	603 (79.8%)		
	I don’t know	103 (13.6%)		
Tumor and cancer are the same thing	False	406 (53.7%)	157.960^a^	0.000
	True	129 (17.1%)		
	I don’t know	221 (29.2%)		
A doctor can tell by looking at an X-ray if a person has cancer	False	370 (48.9%)	153.008^a^	0.000
	True	99 (13.1%)		
	I don’t know	287 (38.0%)		
A biopsy is a procedure to take a small sample of cells	False	29 (3.8%)	304.389^a^	0.000
	True	396 (52.4%)		
	I don’t know	331 (43.8%)		
Cancer treatment is most effective when the cancer has not spread to other parts of the body	False	34 (4.5%)	491.167^a^	0.000
	True	523 (69.2%)		
	I don’t know	199 (26.3%)		
Surgery works best if the cancer is still found at its primary site or where it first started to grow	False	44 (5.8%)	290.032^a^	0.000
	True	420 (55.6%)		
	I don’t know	292 (38.6%)		
The majority of all cancers are caused by a mutation in a gene, which can be passed from one generation to the next in a family	False	125 (16.5%)	123.167^a^	0.000
	True	374 (49.5%)		
	I don’t know	257 (34.0%)		
There can be long periods of time between expose and the appearance of a cancer, sometimes it can take decades	False	68 (9.0%)	247.365^a^	0.000
	True	420 (55.6%)		
	I don’t know	268 (35.4%)		
Some cancers have a genetic component so developing cancer can be a mix of genetics and environment	False	88 (11.6%)	239.460^a^	0.000
	True	434 (57.4%)		
	I don’t know	234 (31.0%)		
Being physically active can help reduce the risk of cancer, improve outcomes after diagnosis, and reduce the risk of cancer returning	False	61 (8.1%)	340.167^a^	0.000
	True	472 (62.4%)		
	I don’t know	223 (29.5%)		
Cancer prevention guidelines recommend eating foods rich in fruits, vegetables, and whole grains, and limiting sugary foods and beverages, red and processed meats, sodium, and alcohol	False	27 (3.6%)	586.341^a^	0.000
	True	554 (73.3%)		
	I don’t know	175 (23.1%)		
Being overweight or obese increases cancer risk	False	167 (22.1%)	52.452^a^	0.000
	True	260 (34.4%)		
	I don’t know	329 (43.5%)		
There is no link between tobacco use and cancer	False	657 (86.9%)	976.341^a^	0.000
	True	50 (6.6%)		
	I don’t know	49 (6.5%)		
Smoking tobacco only causes cancer	False	524 (69.3%)	440.508^a^	0.000
	True	112 (14.8%)		
	I don’t know	120 (15.9%)		
There is no link between alcohol and cancer	False	657 (86.9%)	976.341^a^	0.000
	True	50 (6.6%)		
	I don’t know	49 (6.5%)		
Drinking alcohol can cause cirrhosis, a condition strongly linked to liver cancer	False	58 (7.7%)	327.167^a^	0.000
	True	463 (61.2%)		
	I don’t know	235 (31.1%)		
Ultraviolet (UV) radiation is a known carcinogen that has been directly linked to melanoma and other types of skin cancer	False	33 (4.4%)	448.913^a^	0.000
	True	505 (66.8%)		
	I don’t know	218 (28.8%)		
Only older people get cancer	False	688 (91.0%)	1,132.032^a^	0.000
	True	26 (3.4%)		
	I don’t know	42 (5.6%)		
The HPV vaccine is only recommended for girls	False	136 (18.0%)	596.167^a^	0.000
	True	55 (7.3%)		
	I don’t know	565 (74.7%)		
Having recommended cancer screening exams help to find cancer early when it can be best treated	False	30 (4.0%)	893.556^a^	0.000
	True	638 (84.4%)		
	I don’t know	88 (11.6%)		
Tuibur, khaini, sahdah can cause cancer	False	43 (5.7%)	726.722^a^	0.000
	True	599 (79.2%)		
	I don’t know	114 (15.1%)		
Smoking can cause cancer	False	37 (4.9%)	982.310^a^	0.000
	True	658 (87.0%)		
	I don’t know	61 (8.1%)		
Frequent consumption of smoked food can cause cancer	False	88 (11.6%)	174.770^a^	0.000
	True	377 (49.9%)		
	I don’t know	291 (38.5%)		
Once you’ve had cancer you can never be ‘normal’ again	False	518 (68.5%)	422.508^a^	0.000
	True	106 (14.0%)		
	I don’t know	132 (17.5%)		
A person with cancer is to blame for their condition	False	553 (73.1%)	546.675^a^	0.000
	True	71 (9.4%)		
	I don’t know	132 (17.5%)		
I would feel comfortable around someone with cancer	FALSE	77 (10.2%)	581.387^a^	0.000
	True	460 (60.9%)		
	Blank (unanswered)	183 (24.2%)		
	I don’t know	35 (4.6%)		
More government funding should be spent on the care and treatment of those with cancer	False	27 (3.6%)	1,133.280^b^	0.000
	True	580 (76.7%)		
	I don’t know	141 (18.7%)		
	Blank (unanswered)	8 (1.1%)		
We have a responsibility to provide the best possible care for people with cancer	False	34 (4.5%)	1,080.413^a^	0.000
	True	678 (89.7%)		
	I don’t know	44 (5.8%)		
Cancer treatment can include one or more of the following: surgery, chemotherapy, radiation and hormone therapy	False	36 (4.8%)	442.286^a^	0.000
	True	504 (66.7%)		
	I don’t know	216 (28.6%)		
Secondhanded smoke may bother people, but it isn’t dangerous to their health	False	583 (77.1%)	655.167^a^	0.000
	True	106 (14.0%)		
	I don’t know	67 (8.9%)		
If you have a family member who has been diagnosed with cancer you may need to benign having recommended screening exams at a younger age	False	99 (13.1%)	367.630^b^	0.000
	True	359 (47.5%)		
	I don’t know	270 (35.7%)		
	Blank	28 (3.7%)		
Mammograms help to find breast changes early that may be cancer	False	48 (6.3%)	394.508^a^	0.000
	True	218 (28.8%)		
	I don’t know	490 (64.8%)		
Pap Tests beginning at age 21 for women can find and treat pre-cancerous cells before they become cancer	False	26 (3.4%)	457.365^a^	0.000
	True	226 (29.9%)		
	I don’t know	504 (66.7%)		
Sunscreens have a sun protection factors number that rates their effectiveness in blocking UV rays, one needs to use in winter, summer and in cloudy days	False	48 (6.3%)	780.103^a^	0.000
	True	613 (81.1%)		
	I don’t know	95 (12.6%)		
Benzene and Asbestos are two examples of substances in the environment that can cause cancer	False	38 (5.0%)	571.294^a^	0.000
	True	165 (21.8%)		
	I don’t know	553 (73.1%)		
Radiation therapy is a systematic treatment	False	90 (11.9%)	182.000^a^	0.000
	True	276 (36.5%)		
	I don’t know	390 (51.6%)		
Chemotherapy is a systematic treatment	False	35 (4.6%)	313.310^a^	0.000
	True	425 (56.2%)		
	I don’t know	296 (39.2%)		
Each cancer treatment plan is specific to each person based upon what is the best possible treatment of their specific cancer	False	33 (4.4%)	828.198^a^	0.000
	True	623 (82.4%)		
	I don’t know	100 (13.2%)		
All benign tumor leads to cancer	False	186 (24.6%)	324.627^a^	0.000
	True	91 (12.0%)		
	I don’t know	479 (63.4%)		
There is nothing a person can do to help prevent cancer or find cancer early	False	509 (67.3%)	400.056^a^	0.000
	True	94 (12.4%)		
	I don’t know	153 (20.2%)		
Eating processed foods is a healthy choice	False	345 (45.6%)	52.357^a^	0.000
	True	195 (25.8%)		
I don’t know	216 (28.6%)		
Drinking less water can cause cancer	False	233 (30.8%)	165.579^a^	0.000
	True	118 (15.6%)		
	I don’t know	405 (53.6%)		
Exposure to bacterial and viral pathogens can cause cancer	False	109 (14.4%)	144.437^a^	0.000
	True	270 (35.7%)		
	I don’t know	377 (49.9%)		

Secondhanded smoke: Secondhanded smoke (also known as passive smoke or environmental tobacco smoke) is the involuntary inhalation of tobacco smoke by non-smokers. It comes from: The smoke exhaled by someone smoking (mainstream smoke) and The smoke emitted from the burning cigarette or cigar (sidestream smoke). Superscripts a and b in the table shows the version of the chi-square test used: ^a^Chi-square goodness-of-fit test was used to determine the statistical significance of observed frequencies. A *p*-value of <0.05 was considered statistically significant. ^b^Chi-square test of independence used to determine if there is a significant association between two categorical variables. The very high chi-square value and very low *p*-value mean the distribution of answers is highly significantly different from what would be expected by chance. A *p*-value of 0.000 (usually reported as *p* < 0.001) means the result is highly statistically significant.
